# Exploring Carbon Dots: Green Nanomaterials for Unconventional Lasing

**DOI:** 10.1002/smll.202403653

**Published:** 2024-08-20

**Authors:** Gianluca Minervini, Annamaria Panniello, Carlo Nazareno Dibenedetto, Antonino Madonia, Elisabetta Fanizza, Maria Lucia Curri, Marinella Striccoli

**Affiliations:** ^1^ Institute for Physical and Chemical Processes (IPCF) CNR via Orabona 4 Bari 70125 Italy; ^2^ Department of Physics and Chemistry “E. Segré” University of Palermo Via Archirafi 36 Palermo 90123 Italy; ^3^ Chemistry Department University of Bari via Orabona 4 Bari 70125 Italy; ^4^ National Interuniversity Consortium of Materials Science and Technology INSTM, Bari Research Unit Via Orabona 4 Bari 70125 Italy

**Keywords:** anti‐counterfeiting, carbon dots, fluorescence, lasers, optical cavities, random lasing, sustainable nanomaterials

## Abstract

In recent years, the progress toward lighting miniaturization is focused on luminescent nanomaterials. Among them, fluorescent carbon dots (CDs) are receiving increasing attention thanks to their astonishing optical properties complemented by their intrinsic biocompatibility and low toxicity. The CDs can be easily dispersed in water, organic solvents or incorporated in polymeric matrices, preserving their emission properties. However, the relationship between their structural and optical properties is still not fully elucidated, motivating a consistent research effort for the comprehension of their features. Nevertheless, CDs demonstrate to be efficient gain materials for lasing, thanks to their high quantum yield (QY), emission tunability in the visible and near infrared (NIR) range, short lifetimes, and high absorption cross section, even if the synthetic reproducibility, the low reaction yield and the spectral width of the emission may limit their effective exploitation. This review summarizes the latest advancements in the investigation of the characteristic properties of CDs that make laser action possible, illustrating optical geometries for lasing and random lasing, both in solution and solid state, and the few currently demonstrated breakthroughs. While the journey toward their effective application is still long, the potential of CD‐based laser sources is promising in various technological fields and futuristic perspectives will be discussed.

## Graphical abstract

Carbon dots demonstrate to be an efficient gain material for lasing, due to their chemical structure, outstanding optical properties, and accessible synthetic protocols. This review highlights recent advancements in the field, illustrating mechanisms, optical geometries, and current applications. Despite the challenges in synthesis, carbon dot‐based lasers hold the potential for significant breakthroughs across various technological fields, with promising futuristic implications.

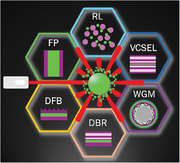

## 1 Introduction

Advancements towards greener, digital, and more automated technologies need materials with enhanced functionalities. Fluorescent materials are ubiquitous in many technological areas, and biomedical imaging, drug delivery, tissue engineering, antimicrobials, (bio)sensors, photocatalysis, photovoltaics, photonics, and optoelectronics, are some notable examples^[^ ^1^, ^2^ ^]^ Innovation in developing fluorescent materials can significantly impact several enabling technologies of the current industrial transition, including healthcare, automated manufacturing, anti‐counterfeiting, information storage devices, degradation of environmental pollutants, and energy‐efficient lighting devices.^[^ ^3^, ^4^, ^5^, ^6^, ^7^ ^]^ In addition, in recent years, the technological demand for miniaturization in advanced devices to minimize energy consumption while simultaneously achieving high efficiency and optimal performance has enormously increased.

In this perspective, nanoscience and nanotechnology are excellent candidates to fill the gap between planning an interconnected and smarter world and realizing it.^[^ ^8^, ^9^, ^10^ ^]^ Especially, among fluorescent materials, luminescent nanoparticles (NPs) can be applied in several applicative areas while adding functionalities and efficiency, and optimizing benefit‐cost ratios.^[^ ^4^, ^11^, ^12^, ^13^, ^14^ ^]^ As an example, fluorescent NPs can improve light‐emitting diodes (LEDs) and display technology.

In this panorama, colloidal quantum dots (QDs) (e.g. CdSe, CdS, PbSe, CdTe, etc.) witnessed a huge global interest in recent years. The recognition of the 2023 Nobel Prize in Chemistry to M. G. Bawendi, L. E. Brus, and A. Yekimov, for their discovery and development, is a robust testimony to this.^[^ ^15^ ^]^ Nowadays QDs find applications in LEDs,^[^ ^16^ ^]^ particularly for display technology, in high color purity lasing^[^ ^17^, ^18^ ^]^ and for biosensor screening products,^[^ ^19^ ^]^ where they are used for the labeling of large biomolecules. Nonetheless, many other applications at the market stage may soon find benefit from their implementation.^[^ ^15^ ^]^

The main concern associated with the use of QDs or lead‐halide perovskite NCs is that they contain toxic and/or polluting elements. The presence of Cd or Pb raises major environmental and toxicological concerns, necessitating serious considerations for regulating the use of these colloidal NPs on the global market scale.^[^ ^20^ ^]^ In such a context, recently Carbon Dots (CDs) emerged as a sustainable and cost‐effective alternative to classical QDs, concomitantly characterized by very intriguing fluorescence properties.

CDs are 0D NPs with a carbonaceous structure characterized by visible‐light fluorescence. Their size is typically 1–10 nm and they are chemically composed of light elements (C, O, H, and eventually N, S, P, B, etc.). In particular, the inner carbon structures can be based on sp^2^ or sp^3^ hybridization. Nonetheless, the optical fluorescence of these NPs is governed by the presence of large polyconjugate and/or polyaromatic domains or by the presence of molecular dyes within the NPs that provide a bright multicolored fluorescence emission.^[^ ^21^, ^22^, ^23^, ^24^, ^25^ ^]^

Classification of CDs based on the structure and composition of their carbonaceous core is very common and widely accepted.^[^ ^26^, ^27^, ^28^, ^29^, ^30^ ^]^ CDs with a core composed of one or a few nanometer‐sized graphene layers are frequently referred to as Graphene Quantum Dots (GQDs). These NPs stand out for having the graphene layer stacking dimension much lower than the other two dimensions, resulting in a distinctive disk shape. In contrast, Carbon Quantum Dots (CQDs) feature multiple ordered graphene layers stacked in the core forming a spheroidal NP. Notably, both GQDs and CQDs exhibit optical fluorescence intricately linked to the quantum confinement effect, albeit with possible contributions from surface state recombination.^[^ ^26^, ^27^, ^29^, ^30^ ^]^ When the carbonaceous core contains both ordered graphitic and amorphous domains, the NPs are commonly denoted as Carbon Nanodots (CNDs). Lastly, Polymer Dots (PDs) are CDs derived from polymeric carbonaceous precursors. Thus, these nanosystems consist of repeating units from polymer or copolymer chains. They may incorporate aromatic groups forming conjugated sp^2^ carbon domains or sub‐fluorophore groups embedded within a cross‐linked polymeric structure.^[^ ^31^, ^32^ ^]^

Besides the core, the CD surface can host small chemical groups with different polarities (‒COH, ‒COOH, ‒NH_2_, ‒CONH_2_, etc.). Often, these groups are inherited or formed by condensation reactions of the molecular carbonaceous precursors employed for CD synthesis. In some other cases, the surface may also contain long aliphatic carbon chains, deriving, for example, from polymers or surfactants added to synthetic mixtures.

Developing CDs with cost‐ and energy‐effective methods and bright fluorescence in the visible range is one of the major scopes of the current scientific effort in the CD area. As a consequence, an outstanding large variety of methods was reported so far enabling their preparation.^[^ ^21^, ^33^, ^34^, ^35^, ^36^ ^]^ It was found that CDs are frequently generated in several chemical or physical processes involving the treatment of carbonaceous matter,^[^ ^34^, ^37^, ^38^ ^]^ namely, when thermal energy or a high chemical oxidation potential is applied to a starting carbonaceous material. The most traditional way to classify these preparative strategies is the division between *top‐down* and *bottom‐up* approaches.^[^ ^39^, ^40^, ^41^, ^42^ ^]^

In *top‐down* methods, CDs are obtained from the reduction of the size of bulk carbon materials (e.g., graphite, candle soot, natural and renewable materials) or larger carbon NPs (e.g., carbon nanotubes, graphene oxide, fullerenes).^[^ ^38^, ^40^ ^]^ The cutting can be achieved by a physical process (e.g., laser ablation, arc discharges) or electrochemical oxidation.^[^ ^41^ ^]^

On the other hand, in *bottom‐up* strategies, molecular precursors are used, and thermal energy in specific synthetic conditions is applied to induce chemical reactions leading to carbonization, forming fluorescent carbonaceous NPs. The organic precursors in these *bottom‐up* approaches are typically selected based on two types of criteria, both regarding their chemical structure and their sustainability. First, it is commonly required that these molecules have the desired chemical reactivity according to specific synthetic needs.^[^ ^21^, ^24^, ^33^, ^37^, ^43^, ^44^ ^]^ Moreover, cost‐effectiveness and abundance of these compounds are key factors to consider for their use in NP chemical syntheses.^[^ ^38^, ^45^, ^46^, ^47^ ^]^ Precursors can be small molecules or macromolecular materials such as polymers, biological macromolecules, or biomass.^[^ ^47^ ^]^ Considering their different chemical structures, molecular precursors comprehend small polar organic molecules,^[^ ^24^, ^43^, ^48^ ^]^ aromatic or polycyclic aromatic compounds terminated by hydroxyl or amino groups,^[^ ^49^, ^50^ ^]^ or other small molecules selected to impart specific properties to the final NPs.^[^ ^51^, ^52^, ^53^, ^54^, ^55^ ^]^

The optical fluorescence of CDs, that can be excitation wavelength (λ_ex_) dependent or independent, as a function of the used precursors and synthetic techniques, is currently ascribed to three main contributes, namely: the formation of sp^2^ domains in the carbogenic core, the radiative recombination at NP surface or defect states, or the presence of molecular fluorophores deriving from the carbonization processes.^[^ ^24^, ^25^ ^]^ In the first case, thermal carbonization of precursor molecules can lead to the formation of poly‐conjugated hydrocarbons, which then become integral constituent parts of the CD carbonaceous core.^[^ ^56^, ^57^, ^58^ ^]^ These π‐conjugated domains can exhibit visible‐range optical absorption and emission, determining CDs’ core photoluminescence (PL). Additionally, excited states of the CDs’ core may emit via surface or defect state recombination.^[^ ^59^, ^60^ ^]^ Moreover, as demonstrated in several reports, fluorescent molecules can form as carbonization intermediates^[^ ^61^, ^62^, ^63^, ^64^ ^]^ and be incorporated within the CDs or adsorbed on their surface, further influencing their overall emission spectra. Often, these three possible mechanisms co‐exist in the same *bottom‐up* synthesized NPs, which gives rise to very complex emission characteristics and recombination dynamics.^[^ ^26^, ^39^, ^41^ ^]^

The heterogeneous luminescence of CDs arising from multiple PL contributions reflects a multi‐level electronic energy structure for these nanoparticles. This intricate energy landscape may, under appropriate conditions, facilitate an effective amplified stimulated emission (SE) regime, making CDs promising candidates for lasing applications. However, this complexity also poses challenges for the rational design of lasing‐suitable CDs and the practical implementation of their spectroscopic characteristics in lasing devices.

**Figure 1:**
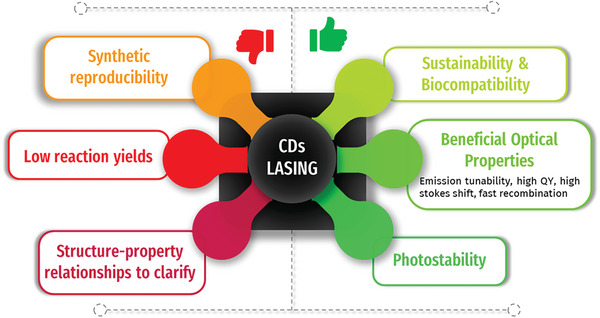
Main drawbacks and benefits of CDs as gain media for lasing applications.

Unlike QDs, for these carbonaceous nanomaterials, the relationship between structure and properties is not easily and unambiguously determined. At the same time, there are so many synthetic parameters and preparatory methodologies (different types of precursors, solvents, temperatures, pressures, purification processes and passivation techniques, surface functionalization, etc.) that even identical syntheses in different laboratories sometimes produce results not always reproducible.^[^ ^65^ ^]^ Another significant concern arises from the low synthetic yields, even with substantial amounts of precursors utilized, often hindering the attainment of highly concentrated solutions. Despite the difficulties related to the synthesis and reproducibility of their morphological, chemical, and physical features, CDs have gained significant attention due to their strong fluorescence, high photostability, biocompatibility, and easy preparative methodologies. They can be dispersed in various solvents or polymer matrices, allowing for solution‐processable fabrication of optoelectronic or sensing devices and facilitating the development of scalable manufacturing processes. While carbonaceous NPs are extensively studied for various applications, such as bioimaging,^[^ ^66^ ^]^ sensing,^[^ ^67^ ^]^ drug delivery,^[^ ^68^ ^]^ and photovoltaics,^[^ ^69^ ^]^ their potential as active materials in lasers is only recently explored. The interest derives from their optical properties, including emission with intense quantum yield (QY), relatively short lifetime, and high absorption cross‐section, tunable by adjusting their synthesis conditions and surface chemistry. In addition, CDs exhibit broadband emission across the visible and near‐infrared spectral regions that can be advantageous for generating laser output at multiple wavelengths or for broadband tunability (**Figure** **1**).

In this review, we will show that, regardless of the synthetic drawbacks and the complex attribution of the emission and recombination mechanisms, all the mentioned properties can effectively translate into stimulated emission of photons and amplification of light. Starting from the pioneering work of W. F. Zhang^[^ ^70^ ^]^ in 2012, the number of papers concerning the use of CDs as active materials increased constantly, demonstrating several examples of lasing systems using nontoxic and biocompatible NPs. In the following, we will discuss properties of CDs synthesized with different preparative techniques and employed for lasing both in solution and at the solid state. Moreover, we will investigate different optical geometries and effective cavities for lasing and random lasing (RL) based on CDs as active gain medium, illustrating several examples present in the literature and the few currently demonstrated breakthroughs.

Despite the efforts of the scientific community in trying to develop CD‐based laser devices with reliable and controlled properties, the road toward standardization is still long, and real applications require the development of advanced technological solutions. However, the prospects are certainly interesting and some indications in this regard will be conclusively reported.

## 2 Solution‐Processed CDs for Lasing

A distinguishing characteristic of CDs is the possibility of dispersing them in various polar solvents thanks to different chemical groups at their surface. Unquestionably, creating stable dispersion of NPs in liquid solvents offers a very simple and effective opportunity to successfully obtain a uniform lasing gain medium. Consequently, CDs prepared with significantly disparate methods were investigated for solution lasing.

In the early stages of CD synthetic research, *top‐down* fabricated CDs were used to demonstrate the first achievement of lasing action in solution.^[^ ^71^, ^72^ ^]^ The CDs were obtained by laser ablation of graphite powder or graphene sheets, and dispersed in a transparent organic solvent, e.g. ethanol (EtOH), or N‐methyl‐2‐pyrrolidone (NMP). The CD solution was then tested as a gain medium to investigate the possibility of achieving optically amplified laser modes.

More extensive research in recent years was devoted to CDs synthesized via *bottom‐up* methods, due to the greater flexibility of the solution‐based synthetic approaches.^[^ ^64^, ^73^, ^74^, ^75^, ^76^, ^77^, ^78^, ^79^, ^80^ ^]^ Through the opportune choice of molecular carbonaceous precursors and preparative protocols, *bottom‐up* routes allow the preparation of CDs, whose properties can be modulated in structure, surface chemical composition, and optical properties.^[^ ^36^, ^43^, ^44^, ^81^, ^82^, ^83^, ^84^ ^]^ Based on solution‐processed protocols, a significative effort was made to relate the CD structural, chemical, and optical features (e.g. crystal structure, surface chemistry and composition, QY, excitation dependence of PL, excited state lifetimes) to SE and lasing parameters, such as laser threshold and narrowing of spectral linewidth.

**Figure 2:**
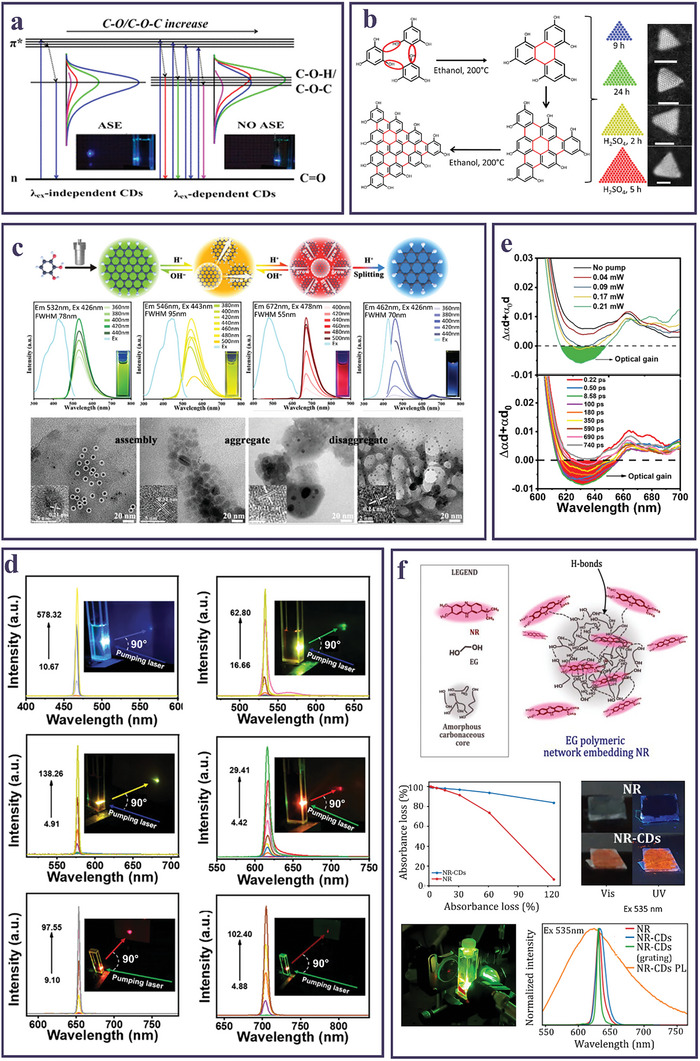
a) Energy‐level diagrams of CDs with excitation independent or dependent PL, correlated to the content of epoxide and hydroxyl surface groups. Reproduced and adapted with permission.^[^ ^78^ ^]^ Copyright 2016, American Chemical Society. b) Scheme of the solvothermal polycondensation of phloroglucinol (PG) leading to multicolor emitting triangular‐shaped CDs.; HAADF‐STEM images of the CDs varying reaction time and synthesis conditions. Scale bars, 2 nm. Reproduced and adapted with permission.^[^ ^88^ ^]^ Copyright 2018, Springer Nature. c) Sketch of the solvothermal reaction of pyrogallol and pH‐dependent transformation process of CDs; photoluminescence excitation (PLE) and PL spectra of CD solution upon acid‐base addition (insets: photographs under UV light.) and corresponding TEM images. Reproduced and adapted with permission.^[^ ^89^ ^]^ Copyright 2023, Wiley‐VCH GmbH. d) Laser emission spectra of the blue, green, yellow, red, deep red and NIR CD solutions prepared by different synthetic approaches at increasing pumping fluences (mJ cm^−2^); insets show the corresponding far‐field laser spots. Reproduced and adapted with permission.^[^ ^79^ ^]^ Copyright 2023,Wiley‐VCH GmbH. e) Optical gain pump‐dependent TA spectra of CDs at ≈1.3 ps delay time and at various delay times. Reproduced and adapted with permission.^[^ ^77^ ^]^ Copyright 2021, Wiley‐VCH GmbH. f) Schematic representation of the structure of NR‐CDs; photobleaching (PB) measurements of NR and NR‐CDs in solution under UV light; pictures of NR and NR‐CD deposited powders under visible and UV light; picture of FP red lasing emission from a NR‐CD solution; SE spectra of NR and NR‐CD solution Reproduced and adapted with permission.^[^ ^64^ ^]^ Copyright CC‐BY 4.0 2023, American Chemical Society.

In their work of 2016, Y. Zhang et al.^[^ ^78^ ^]^ highlighted that in blue‐emitting CDs, the achievement of amplified spontaneous emission (ASE) in solution was correlated with the absence of a marked dependence of the emission from the λ_ex_. The authors synthesized four different types of CDs varying the content of epoxide (C–O–C) and hydroxyl (C–O–H) groups in the NPs: the CDs were prepared either via a *bottom‐up* electrochemical procedure using urea and sodium hydroxide as precursors (CD1) or by a microwave (MW)‐assisted carbonization method using citric acid (CA) and Tris (CD2), L‐cysteine (CD3), ethanolamine (EA) (CD4). Albeit all CDs displayed PL emission in the blue region, only for CD1 and CD3, which displayed an excitation‐independent emission, it was possible to generate ASE, while for CD2 and CD4, exhibiting a PL peak shifting with the λ_ex_, no ASE could be observed even for optical pumping up to 1.35 J cm^−2^. Under optical pumping, CD1 and CD3 showed a narrowing of the PL Full Width Half Maximum (FWHM), down to 4 nm, with a fluence threshold of ≈120 mJ cm ^−2^. This difference in the ASE behavior was correlated to the content of C–O–C and C–O–H groups of CDs inducing localized electronic states below the π* level, as illustrated in **Figure** **2**a.^[^ ^85^, ^86^ ^]^ The high content of these chemical groups was considered responsible for both the excitation‐dependent trend of the PL and the inhibition of ASE. These results were also in agreement with,^[^ ^80^ ^]^ reporting that CDs obtained by MW irradiation of CA and urea and characterized by a near excitation‐independent PL were good candidates for lasing. On the contrary, no laser emission was obtained for CDs synthesized by a similar protocol modifying the precursor mass ratio and displaying a marked excitation dependence of PL emission.

In a subsequent work, Zhang et al.^[^ ^87^ ^]^ hypothesized that the density of localized excited states below the π* level can be tuned by modifying CDs’ surface composition, through controlled carbonization in the NP synthesis, thus helping to achieve population inversion and reducing the laser threshold within a rational synthetic design. Blue‐emitting CDs were synthesized by an MW‐assisted carbonization method. Modulating the molar ratio between the CA and L‐cysteine precursors, CDs with a variable content of 5‐oxo‐3,5‐dihydro‐2H‐thiazolo[3,2‐a]pyridine‐7‐carboxylic acid (TPCA) molecules adsorbed on the NP surface were prepared. These molecules, responsible for CDs’ PL, led to excitation‐independent emission and allowed achieving lasing under optical pumping in a quartz cuvette. The authors demonstrated that the lasing threshold decreases by increasing the CA‐to‐L‐cysteine molar ratio in the range of 1:1 to 2:1, the latter providing the minimum value of 165.5 mJ cm^−2^. This behavior could be correlated to a decrease in the density of localized excited state levels inside the n‐π* gap at a higher precursor molar ratio. Such a lower density of sp^3^‐related excited states facilitated the attainment of population inversion and light amplification, thus demonstrating a viable strategy to reduce the laser threshold of CDs, when the laser action is related to CD surface chemistry.

Eventually, the development of CD synthetic methods, coupled with more accurate isolation and purification strategies, led to the realization of solution lasing from excitation‐independent CDs emitting across the whole visible spectrum. F. Yuan et al.^[^ ^76^ ^]^ utilized triangular‐shaped CDs synthesized starting from phloroglucinol (1,3,5‐Trihydroxybenzene) as the carbonaceous precursor^[^ ^88^ ^]^ to demonstrate RL in a vertically pumped quartz cuvette. The synthesis consisted in a polycondensation process of PG, carried out either in solvothermal or in reflux conditions, and followed by a silica column chromatography purification. By this protocol the authors demonstrated the possibility of controlling the polycondensation by varying reaction parameters, leading to triangular‐shaped NPs with an increasing extension of polyaromatic domains and excitation‐independent emission in the blue, green, and red (Figure 2b). A PG similar precursor, the pyrogallol (1,2,3‐Trihydroxybenzene), was also exploited in a one‐step solvothermal treatment to prepare CDs whose emission tuned from blue to deep red by pH regulation (Figure 2c).^[^ ^89^ ^]^ The as‐synthesized CDs exhibited a green emission, which could be smoothly and reversibly converted into yellow and deep‐red emission by adding a suited amount of acids or alkalis. Reversible NP aggregation, corroborated by TEM measurements, explained the observed fluorescence shifts. On the other hand, the excess addition of acid to the deep‐red CDs led to irreversible disaggregation forming blue‐emitting CDs with a small luminescent core. Next, the authors investigated the possible attainment of ASE in solution with all the CDs emitting in the different spectral regions. Due to the superior features of spectral narrowness, excitation independence, and resonance with pumping beam at 532 nm, only the deep‐red CDs exhibited ASE, observable with a narrowing of the emission spectrum FWHM down to 5 nm for a pumping threshold of 57.7 mJ cm^−2^. In the same year, Y. Zhang et al.^[^ ^79^ ^]^ demonstrated solution lasing of a wide array of CDs synthesized by different *bottom‐up* procedures and exhibiting an excitation‐independent PL in the blue, green, yellow, red, up to reaching the NIR spectral range, where lasing with most common molecular dyes presents several challenges.^[^ ^90^, ^91^ ^]^ The authors correlated the structural, morphological, chemical, and optical characteristics with the measured lasing properties of the optically pumped CDs solutions in a quartz cuvette (Figure 2d), demonstrating that, also for CDs, high QYs and short excited state lifetimes lead to a high radiative decay rate (K_R_), which favors the reduction of the ASE threshold.^[^ ^77^, ^79^, ^92^ ^]^ Moreover, the relatively narrow steady‐state PL spectra facilitated the optical amplification. From the point of view of CD structural properties, the synthesized CDs displayed a low percentage of sp^3^ hybridized carbon and a carbonaceous core with a graphitic crystalline structure. This can be advantageous for the achievement of lasing action, resulting in excited state energy levels that collect many excited electrons with the same energy. In the perspective of identifying suitable energy diagram levels of CDs for an efficient SE, ultrafast transient absorption (TA) measurements can be performed,^[^ ^64^, ^77^, ^79^ ^]^ alongside steady‐state and nanosecond‐timescale resolved PL analyses. As demonstrated in,^[^ ^79^ ^]^ a methodical assessment based on TA spectra allowed to compare the optical properties of the different CDs, emitting over all the visible and NIR spectrum. The displayed negative SE bands, sometimes overlapping with ground state bleaching (GSB) signals, allowed to identify possible lasing spectral regions, with an estimation of delay times and laser threshold. Analogously, Y. Zhang et al.^[^ ^77^ ^]^ analyzed the ultrafast spectroscopic features of red‐emitting CDs solvothermally synthesized by CA and Tris in formamide (FA) as the reaction solvent, in the form of Δαd + α_0_d, where Δα and α_0_ are the transient and steady‐state absorbances, respectively, and d is the excitation optical path (Figure 2e). A negative band, formed at increasing pumping fluence increased, indicated the spectral region of the optical gain. Then, investigation of TA spectra at different time delays, determined the gain time range.

Finally, in the synthetic effort to design CDs with efficient lasing performances, a recent work by our group^[^ ^64^ ^]^ proposed an alternative preparative approach. The developed solvothermal synthetic protocol used a laser molecular dye as precursor and EG as reaction solvent. Specifically, Neutral Red (NR) was selected, thanks to its low toxicity and emission in the red region. Carbonaceous NPs consisting of an amorphous carbogenic matrix embedding NR molecules were synthesized, showing profitable characteristics of stability and PB resistance (Figure 2f). Unlike the other *bottom‐up* synthesized CDs outlined above, the lasing properties of the NR‐derived CDs are predetermined by the optical features of the dye molecules that, after a carbonization process, result incorporated in the carbon nanostructure. Thus, this approach circumvents the necessity to govern the optical properties of CDs through a controlled carbonization during the synthesis process, as the final properties of the CDs derive from the molecular dye used as a precursor, proposing an alternative preparative route especially interesting for achieving CDs with effectively well‐controlled and defined optical and lasing properties.

**Table** **1** presents a thorough examination of both *top‐down* and *bottom‐up* synthetic approaches used to prepare CDs that demonstrated efficient laser action in solution, highlighting their primary optical characteristics.

**Table 1:** Synthetic methods and properties of CDs utilized for lasing in solution.

Synthetic method	Precursors	Solvent	Size [nm]	Surface composition	PL range^a^ ^)^	FWHM PL [eV]	QY [%]	Year^[Ref.]^
Laser ablation	Graphite powder	NMP	1.5–3.5	C═O	B	0.74	n.a.	2012^[^ ^72^ ^]^
Laser ablation	Graphene sheets	EtOH	≈5	C═O, OH	B	0.60	n.a.	2013^[^ ^71^ ^]^
Laser ablation	Graphite powder	EtOH	≈3.5	C═O, OH	B	0.59	n.a.	
MW solution pyrolysis	CA, urea	H_2_O	2–20	C═N, amide, C═O, COOH	G	0.62	18	2014^[^ ^80^ ^]^
Electrochemical solution pyrolysis	Urea, NaOH	EtOH /H_2_O	2	OH, COC, amine	B	0.45	38	2016^[^ ^78^ ^]^
MW solution pyrolysis	CA, L‐Cys	H_2_O	≈2	OH, COC, amine, SH	B	0.49	82	
Electrochemical method*	Graphite rod electrode	H_2_O	≈5	OH, COC, C═O	G	0.51	n.a.	2017^[^ ^93^ ^]^
Electrochemical method	Graphite rod electrode	H_2_O /H_3_PO_4_	≈5	OH, COC, C═O, PO_4_	G	0.77	n.a.	
Hydrothermal method	CDs*, NH_3_	H_2_O	≈5	OH, COC, C═O, NH_2_	B	0.67	n.a.	
Solvothermal/ reflux method	PG	EtOH	1.9	OH	B	0.21	66	2019^[^ ^76^ ^]^
Solvothermal/ reflux method	PG	EtOH	2.4	OH	G	0.18	72	
Solvothermal/ reflux method	PG	EtOH	3.9	OH	R	0.18	54	
MW‐ solution pyrolysis	CA, L‐Cys	H_2_O	3.1	OH, NH_2_, C═O, COC	B	0.62	64.9	2023^[^ ^79^ ^]^
Hydrothermal method	Rhodamine B, NaOH	H_2_O	3.2	OH, NH_2_, C═O, COC	G	0.22	91.2	
Hydrothermal method	Rhodamine B, NaOH	H_2_O	3.8	OH, NH_2_, C═O, COC	Y	0.20	41.2	
Solvothermal method	CA, L‐Cys	FA	3.3	OH, NH_2_, C═O, COC	R	0.26	51.6	
Solvothermal method	o‐PD, BmimPF6	EtOH	4.3	OH, NH_2_, C═O, COC	Deep‐R	0.24	28.3	
Solvothermal method	o‐PD, BmimPF6	EtOH	6.0	OH, NH_2_, C═O, COC	NIR	0.21	37.9	
Solvothermal method	NR dye	EG	5.1	OH, NH, C═N, C—O	R	0.30	9.3	2023^[^ ^64^ ^]^
MW solution pyrolysis	CA, L‐Cys (1:1.25 – 2.25:1)	H_2_O	2.6‐2.8	COOH, SH, amide, CN	B	0.38	51‐83	2023^[^ ^87^ ^]^

^a)^ UV: ultraviolet, B: blue, G: green, Y: yellow, R: red, NIR: near‐infrared.

## 3 CDs in Solid State and Nanocomposites

Solid‐state lasers offer superior performance, reliability, and versatility compared to solution‐based counterparts, making them the preferred choice for many applications in research, industry, and medical fields, thanks to longer operational lifetime, easier handling, and compactness that open the way toward miniaturization.^[^ ^94^ ^]^ Certain fundamental features are essential for CDs to function effectively as durable and reliable active gain material in the solid state. These characteristics include high optical gain, tunable emission wavelength, and long‐term photo and thermal stability.^[^ ^95^, ^96^ ^]^ Specifically, to ensure reliable functionality as gain media, CDs must preserve their optical characteristics under prolonged excitation at high pumping fluence.^[^ ^97^ ^]^ Moreover, most CDs suffer considerable quenching of their fluorescence due to aggregation phenomena in the solid state, severely restricting their use in operating devices.^[^ ^98^, ^99^ ^]^ For this reason, chemical strategies like surface passivation and encapsulation into a matrix, which can limit the aggregation, improve photostability, and increase the QY, are highly desirable.^[^ ^100^, ^101^ ^]^

In this field, polymeric matrices play a crucial role in enhancing the performance and efficacy of CD lasers, primarily by overcoming challenges associated with fluorescence quenching. Indeed, dispersing CDs into a polymeric matrix can prevent their aggregation, also providing a stable and supportive environment for CD gain media, thereby facilitating efficient lasing operations. Moreover, polymers can offer high transparency in the visible region, minimizing light absorption, inducing optical confinement of the emission, and maximizing the output of laser light. Mechanical stability and flexibility, provided by polymeric matrices, are essential for maintaining the structural integrity of CD lasers. Finally, these matrices exhibit resilience to harsh environmental conditions, including high temperatures and corrosion, ensuring high performance and longevity across diverse operational settings.

**Figure 3:**
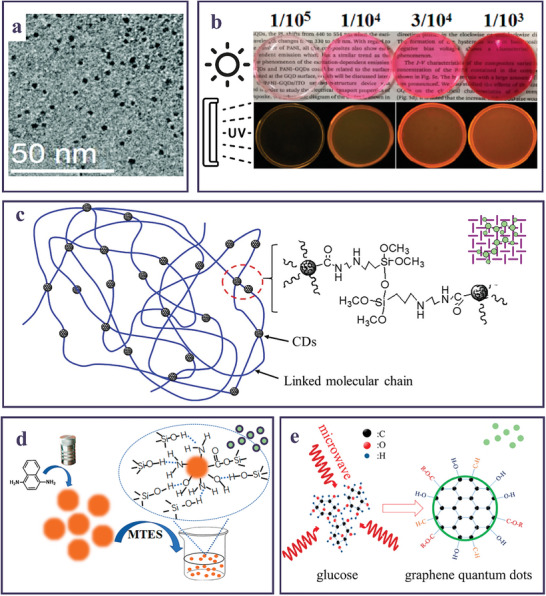
a) TEM image of “in situ” synthesized CDs in presence of a polymeric matrix. Reproduced and adapted with permission.^[^ ^104^ ^]^ Copyright 2017, The Royal Society of Chemistry. b) Pictures of “’ex situ”’ fabricated CD‐gel under sun and UV light at increasing CD loading. Reproduced and adapted with permission.^[^ ^105^ ^]^ Copyright CC‐BY 4.0 2017, MDPI. c) Sketch of CDs interconnected by organosilane chains. Reproduced and adapted with permission.^[^ ^104^ ^]^ Copyright 2017, The Royal Society of Chemistry. d) CD‐ormosil gel nanocomposite preparative steps. Reproduced and adapted with permission.^[^ ^105^ ^]^ Copyright CC‐BY 4.0 2021, MDPI. e) MW‐assisted pyrolysis of glucose to achieve GQDs employed as a powder gain medium. Reproduced and adapted with permission.^[^ ^106^ ^]^ Copyright 2019, American Chemical Society.

The integration of the CDs into a polymeric matrix can be achieved either during the NP synthesis (“in‐situ” process) or afterward, blending the pre‐synthesized nanofillers and the polymer to achieve a nanocomposite (“ex‐situ”).^[^ ^102^ ^]^ In the former case, two main strategies can be identified. One is based on the introduction of monomers in the synthesis batch of the CDs, followed by polymerization to form a nanocomposite, while the second consists in directly adding the polymer during the reaction. These “in situ” procedures promote a thorough dispersion of the CDs in the matrix, leveraging the available active binding sites, as shown in **Figure** **3**a. However, such an approach lacks precise control over the concentration of dispersed CDs in the polymer host. In contrast, the “ex‐situ” method addresses this limitation, enabling the optimization of the active material for specific applications. This is evidenced in Figure 3b, where a high loading of CDs was successfully incorporated in a gel matrix, still preserving high transparency of the nanocomposites.

One of the pioneering method to synthesize “in situ” composites was introduced by Zhang et al.^[^ ^70^ ^]^ who produced PEG200 modified CDs using laser ablation through a Nd:YAG laser (1064 nm, ≈6ns, 10Hz, peak power density ≈65 MW cm^−2^, 30min) onto a mixture of graphite powder and PEG_200_. The nanocomposite exhibited a relevant PL enhancement and laser activity only after a heating treatment of at least 2h at 80°C. In the presence of the hydroxyl groups of the PEG_200_, the thermal process induced the esterification reaction of the carboxylic groups on the surface of the CDs derived from the synthesis. The increase of PL intensity, resulting in enhanced radiative recombination, was ascribed to the better passivation of the CD surface, leading to an effective delocalization of π‐electron.

Besides physical approaches, such as the laser ablation, wet chemistry can also provide “in situ” nanocomposites, as demonstrated by applying a hydrothermal treatment of ε‐poly‐L‐lysine and CA, finally resulting in pure blue light‐emitting polymeric CDs (PCDs).^[^ ^103^ ^]^ Such highly luminescent PCDs consisted of a spongy, amorphous polymeric structure composed of tiny clusters uniformly distributed, incorporating fluorescent aromatic moieties devoid of graphitic carbon. The polymeric framework acted as an optically transparent matrix and effectively protected the emitting moieties from the self‐quenching of PL in the solid state.

The chemical reactivity of some kinds of monomers was also manipulated to design and fabricate “in situ” composites where additional functionalities deriving from the polymeric components were imparted to the resulting material. In this regard, Ni et al.^[^ ^107^ ^]^ took advantage of the chemical reactivity of polystyrene (PS), expanding the concept of “in situ” synthesis and proposing an intriguing approach to simplify the synthesis of CD‐based nanocomposites. In their work, PS acted both as CD precursor and polymeric embedding matrix in a single‐step partial thermal decomposition process. The authors demonstrated a precise control over the concentration of CDs within the PS matrix by varying the reaction time of the synthesis, performed at 340 °C for 20, 25, 30, or 35 min. Afterward, optical fibers were fabricated drawing the melted nanocomposite mixture with a metallic tip and controlling the fiber's width adjusting the pulling rate. Similarly, silanes and their organic derivatives were fruitfully exploited in solvothermal treatments to gain solid‐state CDs. Specifically, N‐(β‐aminoethyl)‐γ‐aminopropyltrimethoxy‐silane (A‐112.0), which facilitates the effective self‐solidification of CDs by linking organosilane chains (Figure 3c).^[^ ^104^ ^]^ The net effect on the optical properties was to enlarge the extension of the π conjugation and form a donor–π‐donor structure, improving the efficiency of multiphoton absorption up to four photons at room temperature. Lasing emission up to three‐photon excitation was achieved from these organosilane functionalized CDs sandwiched between a quartz substrate and a dielectric mirror. Similarly, Wang et al.^[^ ^108^ ^]^ used triethoxyvinylsilane (KH151) as silane precursor, 1,3,5‐benzenetricarboxylic acid (H3BTC) as CDs precursor, and 1,3,5‐benzenetricarboxylic acid trimethyl ester (Et3BTC) as polymeric matrix precursor, in the solvothermal synthesis for the formation of fluorescent hybrid crystals. This approach led to the fabrication of silane functionalized CDs (SiCDs) homogeneously incorporated in Et3BTC solid crystal, with multicolor RL action spanning from near UV to red region.

Conversely to the “in situ” methods, “ex situ” nanocomposite fabrication allows for precise control and optimization of each component's properties, as the synthesis and incorporation into the host matrix occur in separate steps. Moreover, such an approach enables the integration of photoactive nanoparticles into a wide variety of host matrices, including epoxy resins, thermoplastic polymers and ormosil gels. In one of the earlier examples of the “ex situ” method for nanocomposite preparation, the silanolic CDs were integrated in an epoxy matrix to build a gain medium.^[^ ^109^ ^]^ The SiCDs synthesis was performed pyrolyzing CA at 230–260 °C in the presence of a silane coupling agent, namely A‐2120 (N‐(β‐aminoethyl)‐γ‐aminopropylmethylbimethoxy‐silane). The silane provides methoxy groups on the CD surface, making the carbonaceous NPs perfectly integrable in the epoxy resin to obtain a profitable gain medium in the lasing device. Indeed, the authors fabricated a sort of optical fiber guide that exploited the difference in refractive index between the pure epoxy and a fused silica capillary filled with CDs and epoxy, to optically confine the light.

More recently, CDs were synthesized by a solvothermal approach using an ethanol solution of 1,4‐diaminonaphthalene as a precursor, and incorporated in an ormosil‐based gel (Figure 3d).^[^ ^105^ ^]^ Similarly, in another work, the same authors employed 1,3‐dihydroxy naphthalene as the primary source for producing CDs that were dispersed in an epoxy composite.^[^ ^110^ ^]^ In both cases, the synthesized CDs exhibited a QY exceeding 60% in the red region.

The previous approaches involve the incorporation of luminescent CDs in a matrix, easier to handle than solution systems, to enhance their stability and performance for lasing action. However, these strategies can be costly, introduce preparation challenges, and require additional steps in device fabrication. Alternatively, very few examples are reported in literature where CD powders were directly employed as active gain media, yielding promising results.

Lee et al.^[^ ^106^ ^]^ synthesized GQDs pyrolyzing glucose molecules through MW irradiation heating (Figure 3e). Then, the synthesized CD solution was manually drop‐cast to form a film on the top surface of a Ta_2_O_5_/SiO_2_ dielectric distributed Bragg reflector (DBR). This high‐efficiency cavity enabled the realization of a stable and efficient lasing device.

In **Table** **2**, a comprehensive overview of both nanocomposite and powder methodologies utilized to effectively synthesize CDs for solid‐state lasing applications is reported.

**Table 2:** Synthetic methods and properties of CDs utilized for lasing at solid state.

Synthetic method	Precursors	Solvent	Size [nm]	Surface composition	PL range^a^ ^)^	FWHM PL [eV]	λ_ex_ dep^b^ ^)^	QY [%]	Year^[Ref.]^
Laser ablation	Graphite powder	PEG_200_	3.5	C═O, OH, COOH	B	0.31		n.a.	2012^[^ ^70^ ^]^
Laser ablation	Graphene sheets	EtOH	≈5	C═O, OH	B	0.55		n.a.	2013^[^ ^71^ ^]^
Solution thermal pyrolysis	CA	Silane A‐2120	2.9	COOH, SiOC, amide, SiCH_3_	B‐ R	0.51	✓	65‐70	2014^[^ ^109^ ^]^
Electrochemical solution pyrolysis	Urea, NaOH	EtOH /H_2_O	2	OH, COC, amine	B	0.45		38	2016^[^ ^78^ ^]^
MW solution pyrolysis	CA, Urea	H_2_O	≈5	OH, NH_2_, C═O	G	0.40	✓	n.a.	2017^[^ ^111^ ^]^
Solution thermal pyrolysis	CA	Silane A‐112.0	2–4	COOH, Si‐O‐Si	B‐G	0.56	✓	52	2017^[^ ^104^ ^]^
Solvothermal method	DAN	EtOH	2–5	COOH, NH_2_, heterocyclic N	O	0.17		82	2019^[^ ^112^ ^]^
MW‐assisted hydrothermal	Glucose	H_2_O	≈5	OH, COR	G	0.83	✓	∼30	2019^[^ ^106^ ^]^
Solvothermal method	CA, Tris, PEG_400_	DMF	2.5	OH, NH_2_, C═O	R	0.45		65.5	2021^[^ ^77^ ^]^
Solvothermal method	DAN	EtOH	<10	OH, C═O	R	0.22		63	2021^[^ ^105^ ^]^
Direct thermal decomposition	PS	N/A	3.5	PS‐related groups	B	0.35	✓	20	2021^[^ ^107^ ^]^
Solution thermal pyrolysis	DAN, H_2_SO_4_	EtOH	5.2	OH, C═O	R	0.30	✓	66.7	2021^[^ ^110^ ^]^
Solvothermal method	H3BTC, KH151	EtOH	7.6	Si‐O—C	B‐R	1.06	✓	21	2022^[^ ^108^ ^]^
Hydrothermal method	CA, ε‐poly‐L‐lysine	H_2_O	4‐10	OH, NH_2_, COOH	B	0.39		49	2023^[^ ^103^ ^]^
Solvothermal method	Pyrogallol	DMF	≈5	NH_2_, C═O	R	0.64		16	2023^[^ ^89^ ^]^
Solvothermal method	Silane KH151	EtOH	3.5	OH, Si‐O—C	UV – B	0.83		n.a.	2023^[^ ^113^ ^]^
Heat‐induced self‐foaming	PDA, H_3_BO_3_, Urea	N/A	3.2	C═O, O—C═O	G	0.59		≥95	2024^[^ ^114^ ^]^

^a)^ UV: ultraviolet, B: blue, G: green, O: orange, R: red

^b)^ CDs exhibiting λ_ex_‐dependent PL are highlighted with a checkmark.

## 4 Optical Cavities for Carbon Lasing

Commonly, a laser consists of three primary components: an active material responsible for optical gain via SE, an optical or electrical pumping system, and an optical cavity that partly confines and resonates the light. Then, the proper design of an optical cavity is of paramount importance for moving from light emission to lasing, both in solution and in the solid state. The optical cavity is conceived to provide feedback to the gain medium, stimulating the emission of coherent light. Typically, optical cavities consist of two or more highly reflective mirrors facing each other, with one mirror partially transparent to allow a fraction of the laser light to escape outside. The light, generated within the gain medium by proper excitation processes, travels back and forth between the mirrors, undergoing multiple reflections. This repeated bouncing causes the light to resonate within the cavity, amplifying in intensity with each pass. The mirrors are engineered for very high reflectivity, typically exceeding 99%, to ensure maximal reflection back into the cavity. Moreover, the length of the optical cavity is crucial, dictating its resonant frequencies. Indeed, laser light behaves as a stationary wave that only resonates at specific wavelengths determined by the cavity length and by the properties of the mirrors, according to the relation L = nλ/2, where L is the cavity length, λ is the lasing wavelength and n is an integer number. The resonant frequencies correspond to the allowed modes of the cavity, governing the emitted laser light's wavelengths.

The performance of a laser cavity is typically measured by a dimensional parameter known as the Quality (Q) factor, which is calculated as the ratio of the laser line's peak wavelength to its full width at half maximum. The Q‐factor is the ratio of energy stored in the laser cavity to energy lost every cycle owing to absorption, scattering, and light leakage from the cavity. A greater Q‐factor indicates less energy losses and improved light confinement within the cavity, resulting in more efficient laser operation and higher laser output power.

By controlling the properties of the optical cavity, such as the mirror reflectivity and the cavity length, it is then possible to maximize the Q‐factor and tailor the characteristics of the laser beam, including its wavelength, coherence, and spatial properties. Different types of laser cavities can be designed to achieve peculiar properties and performance characteristics and, to compare the efficiency of the optical cavities, specific parameters, such as pump threshold, Q‐factor, signal‐to‐noise ratio, divergence of the beam, etc. can be used.

In the next, we will try to rationalize the literature on laser emission in CD‐developed optical cavities, both in solution and in solid state, making an overview of the more used geometric configurations of the cavities, moving from the classic Fabry‐Perot (FP) type cavities to Vertical‐Cavity Surface‐Emitting Laser (VCSEL) structures, also including the random lasing (RL) and whispering gallery microresonators (WGM), underlining their different properties and the advantages brought by CDs.

### 4.1 Fabry‐Perot laser

The FP cavity is one of the simplest types of laser cavities, consisting of two parallel mirrors, typically flat and highly reflective, facing each other. In the easier FP geometry, the amplification of spontaneous emission in CD solutions can be measured in standard quartz cuvettes,^[^ ^64^, ^75^, ^76^, ^77^, ^78^, ^79^, ^80^ ^]^ focusing a pumping beam onto the emitting solution in the cuvette and detecting the radiation transversally to the incident pumping beam, often using optical fiber detectors. Optical pumping, frequently carried out by a nanosecond pulsed laser source of appropriate wavelength, can be performed either laterally, i.e. focusing the light on a face of the quartz cuvette, or vertically, i.e. from the top side of the cuvette. The optical cavity formed by spherical or flat mirrors, or by a reflection grating, transforms the SE in an effective lasing action, demonstrated by an appreciable decrease of the lasing threshold and a significant narrowing of the laser linewidth. However, due to the difficulty of alignment of the mirrors, this geometry is usually employed for preliminary testing of the gain media on a lab scale, while reducing the mirror separation distance, e.g. to L < 1 cm, the configuration problems are strongly reduced. Plane‐parallel resonators are commonly used in microchips, microcavity lasers, and semiconductor lasers. In these cases, rather than using separate mirrors, a reflective optical coating may be directly applied to the laser medium itself.

**Figure 4:**
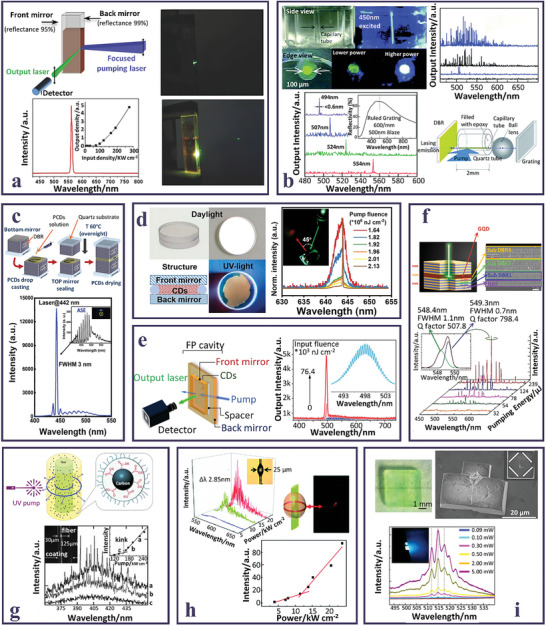
a) Scheme of a Fabry Perot cavity for CD in solution; pictures of the CD‐laser operating at two different pumping densities (λ_ex_ = 355 nm); emission spectrum recorded at 274 kW cm^−2^ (inset: PL peak intensity versus pumping density). Reproduced and adapted with permission.^[^ ^80^ ^]^ Copyright 2013, WILEY‐VCH Verlag GmbH & Co. KGaA, Weinheim. b) Pictures of the capillary tube embedded with CD in epoxy before and after excitation at 450 nm; lasing spectra of the FP CDs‐based laser under different power densities; emission spectra and scheme of a CDs based DFB laser, formed by a grating and a ball lens (inset: reflection spectrum of the grating). Reproduced and adapted with permission.^[^ ^109^ ^]^ Copyright 2014, The Royal Society of Chemistry. c) Scheme of the fabrication steps to obtain a sandwiched DBR cavity containing polymeric CDs; laser emission of polymeric CDs (inset: ASE spectrum under UV pumping). Reproduced and adapted with permission.^[^ ^103^ ^]^ Copyright 2023, American Chemical Society. d) Photographs under day and UV light, and scheme of CDs‐based planar microcavity laser device; normalized PL spectra of the device under different pumping fluences (inset: photograph of the device excited at 532 nm by ≈2 mJ cm^−2^ pumping fluence). Reproduced and adapted with permission.^[^ ^77^ ^]^ Copyright 2021, Wiley‐VCH GmbH. e) Scheme of a FP parallel micro‐resonator for CDs aqueous solution; Emission spectra of the CD‐based laser at various pump fluences (inset: high‐resolution laser emission spectrum at 64.2 µJ cm^−2^). Reproduced and adapted with permission.^[^ ^114^ ^]^ Copyright 2024, Wiley‐VCH GmbH. f) Scheme and SEM image of GQDs‐VCSEL device, formed by sandwiching GQDs in powder between two DBRs; PL spectra of the device at various pumping energies (inset: deconvolution of lasing peak). Reproduced and adapted with permission.^[^ ^106^ ^]^ Copyright 2019, American Chemical Society. g) Schematic illustration of a CD‐PEG surface coated optical fiber acting as a WGM cylindrical microcavity; PL spectra of the device under pumping at 266 nm (inset: laser threshold curve). Reproduced and adapted with permission.^[^ ^70^ ^]^ Copyright 2012, WILEY‐VCH Verlag GmbH & Co. KGaA, Weinheim. h) Lasing spectra of a CDs‐based WGM cylindrical microcavity at different excitation powers (inset: optical microscope picture of the microcavity having a diameter of 25 µm; plot of output intensity versus excitation power of the microcavity (inset: scheme and photograph of the WGM laser). Reproduced and adapted with permission.^[^ ^112^ ^]^ Copyright 2019, The Royal Society of Chemistry. i) Picture of hybrid CDs‐NaCl crystals under UV light; SEM image of the grinded hybrid crystal (inset: resonant pathways in the cubic crystal); PL spectra of a hybrid crystal under different pump powers (inset: photograph of a hybrid crystal under light excitation). Reproduced and adapted with permission.^[^ ^111^ ^]^ Copyright 2017, American Chemical Society.

A first example of an FP cavity laser based on CDs was reported in 2014 by Shen,^[^ ^106^ ^]^ where green emitting carbon NPs in a water‐ethanol mixture, with extended conjugation, high QY, and low overlap between absorption and emission, were used as active media in a quartz cuvette, on the walls of which two mirrors with a reflectance of 99.5% and 95.3% were deposited at a distance of ≈5 mm (**Figure** **4**a). The cavity was pumped using a Nd:YAG laser beam (λ = 355 nm, pulse width 10 ns at a 1‐Hz repetition rate) and focused by a cylindrical lens into a 0.4‐mm ‐wide stripe. The carbon NP solution exhibited a well‐defined beam of bright green polarized laser emission in a direction orthogonal to the mirrors, with measured far‐field divergence less than 2 mrad. Due to the length of the cavity, the laser peak was an envelope of several resonant modes, as expected in an FP resonant cavity. Additionally, these carbon NPs displayed a superior photostability compared to conventional green laser dyes under the same irradiation conditions, a clear advantage when considering laser gain media.

One of the main challenges in the field of CDs is to obtain carbon nanoparticles displaying a red emission with high QY.^[^ ^115^ ^]^ Only in recent times red lasing was demonstrated in an FP cavity: a highly concentrated dispersion of NR‐based CDs in ethanol was excited in a long homemade resonant cavity pumped by an external tunable laser to obtain red lasing at 630 nm.^[^ ^64^ ^]^ The CD precursor was a dye laser incorporated in the carbonaceous matrix, ensuring an energy level configuration suitable to achieve laser emission in NPs which displayed an higher photochemical stability compared to the original dye used as precursor. The best lasing efficiencies were obtained by pumping at wavelengths closer to the absorption maximum. Both longitudinal and transversal geometries were tested, without significant differences in optical performance.

Facing the same difficulties discussed above, only recently lasing in the red region was obtained also in solid state by using a FP planar microcavity; this device was obtained by sandwiching a red‐emitting CD/epoxy film between an Al mirror and a quartz substrate, with the gain film cross‐section of 22 µm.^[^ ^102^ ^]^ A second harmonic of a Nd:YAG laser at 532 nm with a 6 ns pulse width was used to achieve population inversion in the cavity. The laser beam was focused onto the gain medium at an angle of 45° to the mirror's plane and emission light was collected at different angles by an optical fiber. Lasing was achieved when the pump power exceeded 13 kW cm^−2^. However, all the lasing modes were randomly distributed over the emission spectra, due to the scattering of light by inhomogeneous distribution of refractive indices in the gain medium. By increasing the temperature of the laser cavity up to 250 °C, the authors demonstrated that the cavity still supported lasing, transforming from random to directional FP lasing. Indeed, in an FP cavity, the relationship between the laser mode spacing and the optical path can be written as Δλ = λ^2^/2nL, where Δλ is the mode spacing of two adjacent laser modes, λ is the wavelength of the lasing emission, n is the refractive index and L is the thickness of the film (i.e. laser cavity length). The mode spacing at high temperatures was consistent with the calculated values, proving the mode transition and supporting the possible use of CD active materials in laser devices at high temperatures. This stability is likely due to the exceptional ability of carbon NPs to retain their optical properties under such conditions.

One of the main drawbacks hindering the advancement of CDs‐based solid‐state devices is the aggregation induced quenching of carbon NPs.^[^ ^98^, ^99^ ^]^ Compared to liquid dispersions, solid‐state CD aggregates show either lower QYs or, in the worst case, no fluorescence at all. This issue invariably results in higher lasing thresholds for CDs‐based lasers. To overcome this issue, superior solid‐state matrices for CDs have been purposefully developed. For instance, a nanocomposite obtained by uniformly dispersing CDs in an epoxy matrix was used to fabricate a solid‐state laser. The active medium was sandwiched between two reflectors to form a FP cavity.^[^ ^109^ ^]^ Specifically, the CDs/epoxy nanocomposite was placed inside a narrow capillary tube (2 mm in length and 100 µm in inner diameter), which was then enclosed within a larger quartz tube filled with epoxy. This setup provided better optical confinement of the laser emission, due to the epoxy's higher refractive index. The cavity was completed by two external mirrors, an Al mirror (Al‐coated glass substrate) and a DBR mirror (dielectric mirror on a quartz substrate), to increase the Q‐factor of the cavity minimizing the pump threshold (Figure 4b). The use of organosilane groups on the CD surface notably lowered the laser threshold compared to CDs functionalized with carboxyl, ester, or hydroxyl groups. This improvement was attributed to the high degree of functionalization of the CDs surface, which improved the dispersion capabilities of the silanol moieties in the epoxy matrix, preventing CDs’ aggregation in solid state and thereby avoiding emission quenching, as previously discussed.^[^ ^70^, ^109^ ^]^

Similarly, CDs in an ormosil gel matrix were used as the gain medium in a planar cavity.^[^ ^105^ ^]^ It was shown that this matrix enabled SE to dominate over the spontaneous emission at a pump power of 70 W cm^−2^. This resulted in an exceptionally low lasing threshold, attributed to the high PL efficiency of the CDs‐ormosil gel and the twofold optical confinements induced by the microresonator.

### 4.2 DFB, DBR, and VCSEL Cavities

Although the FP cavity is undeniably the simplest configuration to achieve lasing, CDs have successfully demonstrated to be efficient gain media in other kinds of cavities.

An interesting configuration of the laser cavity is named DFB (Distributed FeedBack cavity), which includes a periodic grating structure within the gain medium itself. The grating acts as both the feedback mechanism and the wavelength‐selective element, resulting in single‐mode operation. DFB lasers are widely used in telecommunications due to their narrow linewidth and stable single‐frequency output. In,^[^ ^109^ ^]^ with the purpose of exploiting the wide PL bandwidth of CDs, the authors realized a CD/epoxy nanocomposite designed tunable laser in the visible range; in this work, a Littrow configuration was set up replacing the higher reflectance mirror with a grating coupled to the capillary tube via a ball lens (Figure 4b). In this case, the resonant emission wavelength depends on the angle between the surfaces of the capillary tube and grating (i.e., Littrow angle). Therefore, by changing only a few degrees the grating orientation a wavelength tuning across 60 nm in the visible spectral range was achieved.

As the large PL bandwidth of CDs can lead to poor gains due to competing emission processes, different cavity configurations tuned to specific wavelengths can enhance the lasing performances of the system. DBR lasers utilize Distributed Bragg Reflectors on either side of the gain medium. These reflectors consist of alternating layers of dielectric materials with different refractive indices, forming a periodic structure with repeated pairs of quarter‐wave thickness, acting as high‐quality mirrors at specific wavelengths. DBR lasers allow for precise control of the cavity length and wavelength, making them suitable for applications requiring tunability and narrow linewidth, such as spectroscopy and optical communications.

Powdered PCDs were deposited on the surface of a sputtered DBR mirror, sandwiched with the second DBR mirror, and subjected to a thermal treatment to solidify the gain medium (Figure 4c).^[^ ^103^ ^]^ Since the spectral response of the DBR resonator was purposefully tuned to the PCD emission, an intense lasing peak with a FWHM of 3 nmwas obtained.

Lu et al. prepared a single‐mode DBR micro‐resonator exploiting, as the gain medium, highly red emissive passivated CDs.^[^ ^77^ ^]^ The laser device was a sandwich structure composed of two high reflectivity DBR mirrors in the red, separated by a discontinuous and very thin spacer layer on the border, constituted by silica microbeads of just 300nm. The CDs in solution were injected in the free space among the mirrors and the output emission was detected orthogonally to the front mirrors. As the pump fluence increased, the spectrum suddenly narrowed, and laser emission in the red with a FWHM of only 0.14 nm was obtained, with an impressive Q‐factor of ≈4600 and a single longitudinal mode, thanks to the very short length of the laser cavity (Figure 4d).

Recently, the synthesis of Perylene‐3,9‐dicarboxylic acid (PDA) based CDs by a heat‐induced self‐foaming process in ambient pressure, resulted in novel water‐quenching resistant carbonaceous nanostructures, formed by cross‐arranged sp^2^ domains.^[^ ^114^ ^]^ The hydrophobicity of the CDs is reflected in minimal luminescence quenching caused by interactions with water molecules, leading to near unit QY in aqueous solutions. These highly green luminescent CDs were tested for lasing using a few hundred‐length micro‐resonator with DBR mirrors, properly designed to narrow the reflectivity range to a few tens of nanometers, resulting in a well‐defined beam of bright green laser emission in a direction orthogonal to the mirrors (Figure 4e). The work demonstrated that the CDs’ water dispersions can be used as gain media to achieve lasing in aqueous media, thus opening a promising possibility for CD‐based cellular laser applications.

Finally, the VCSELs have a very compact cavity structure, with the mirrors situated above and below the gain medium. Light emission occurs perpendicular to the surface of the semiconductor chip, resulting in a highly circular and symmetric beam, in low threshold current and efficient power conversion. In addition, the high manufacturability and integrability make them widely adopted in the optoelectronic technology and industry. Compared to the external FP cavity geometry, the monolithic integration of CDs within a VCSEL is conceptually novel, and it is experimentally challenging to realize, as it has a much shorter cavity length to induce the light amplification and to provide sufficient gain for lasing action. The work of Lee demonstrated for the first time the lasing action of graphene CDs in a VCSEL structure obtained by clamping two high reflectivity DBR mirrors, resulting in a cavity length of only a few micrometers (Figure 4f).^[^ ^106^ ^]^

### 4.3 WGM

The first example of laser action in a CD‐based active medium used the whispering gallery micro‐resonator as an optical cavity.^[^ ^70^ ^]^ The microresonators are made of high‐refractive‐index materials, circular in shape, and light diffuses around the resonator's perimeter, generating a steady standing wave pattern, confining light within with minimal loss. WGMs offer various advantages for laser applications. They provide low threshold lasing due to their high Q‐factor. Furthermore, their small size enables for compact and integrated laser sources, making them ideal for use in sensing and on‐chip photonics. The lasing features of WGMs can be adjusted by varying the resonator size, shape, material properties, and coupling arrangements. In,^[^ ^70^ ^]^ a layer of PEG passivated CDs of defined thickness coated an optical fiber. Increasing the power of the pumping beam into the fiber, sharp peaks emerged from the emission spectra of CDs, and a Q‐factor larger than 1300 was calculated, limited by the roughness of the coating/fiber interface (Figure 4g). Furthermore, the lasing mechanism of the cylindrical micro‐cavities was attributed to light partially transmitted through the coating/fiber interface. In 2019, the same authors prepared high QY orange emitting CDs, thanks to the high content of carboxyl groups, and used these NPs as a gain medium in a WGM laser (Figure 4h).^[^ ^112^ ^]^ Modifying the diameter of the fiber, a minimum linewidth of 0.16 nm was demonstrated with a Q‐factor of ≈3600 and a laser threshold of one order of magnitude lower than the previous result. The lasing emission was due to the formation of a close‐loop path for light near the surface of the microcavity, and tunable mode spacing of the laser was demonstrated to be rationally modulated by changing the diameter of the laser cavity.

Finally, WGMs were also evidenced using an original millimeter‐sized cavity obtained by the crystallization of NaCl, incorporating green‐emitting CDs.^[^ ^111^ ^]^ The embedding in NaCl crystals limits CD aggregation‐induced self‐quenching phenomena, also conferring good thermal and long‐term stabilities. The lasing in the CD−NaCl hybrid crystals was demonstrated by the appearance of sharp peaks with narrow widths upon increasing the pump power, and the mode spacing agreed with the simulation for WGM resonators (Figure 4i).

### 4.4 Random Lasing

Contrary to conventional lasers, where highly monochromatic and coherent radiation is achieved by a well‐designed optical cavity that determines the laser modes, in RL light amplification is obtained by multiple scattering within a disordered gain medium, without reflection mirrors.^[^ ^116^, ^117^, ^118^ ^]^ The scatterers typically consist of nanostructured powdered particles dispersed with the active medium material, so that the light emitted following the excitation produced by a pump laser can be diffused and amplified through the disordered medium via multiple events. The average distance travelled by light between two consecutive scattering events, defined as scattering mean path, is one of the key factors in the RL process and it is inversely proportional to the number density and the scattering cross‐section of the scattering particles.^[^ ^116^, ^118^ ^]^

Compared to conventional lasers that require precise design and expensive optics for developing the optical microcavities, RL systems offer lower production costs and simpler technological processes for their fabrication, with a further benefit of the large availability of the scatterers and the high emission efficiency of the active materials allowing for the large‐scale manufacturing. However, despite such undeniable advantages, due to the randomness of the multiple scattering and high energy loss, improvements in the engineering of RL devices are still necessary for achieving controlled spectral emission ranges, optimized output lasing modes, and low pumped thresholds.

In recent times, thanks to their intriguing optical properties, CDs have been applied also in RL processes as viable gain media.^[^ ^73^, ^75^ ^]^ Indeed, CDs can be considered highly disordered nanosystems that can be converted into a mirrorless virtual cavity through multiple light scattering.^[^ ^126^ ^]^

The first application of CDs in RL was reported in 2013 by H. Zhu et al., who achieved RL from oxygen‐containing groups functionalized carbonaceous nanoparticles prepared by laser ablation.^[^ ^71^ ^]^ The authors demonstrated a better laser gain performance in the blue spectral range reached by using graphene as starting material compared to that obtained by using similar CDs prepared from graphite precursor. In such conditions, the carbon nanostructures served as active gain and scattering media. The RL lasing cavity consisted of a quartz tube filled with a GQD or CD ethanol dispersion that was perpendicularly excited by a horizontally polarized pulsed Nd:YAG laser operating at 10 Hz. The concentration of GCDs and CDs was tuned to obtain maximum emission efficiency, evaluated by the variable stripe length method by measuring the net optical gain as a function of the spectral wavelength. The fivefold higher optical gain per pump power observed in GQDs with respect to CDs was attributed to the larger density of functional groups decorating the large GQDs surface.

**Figure 5:**
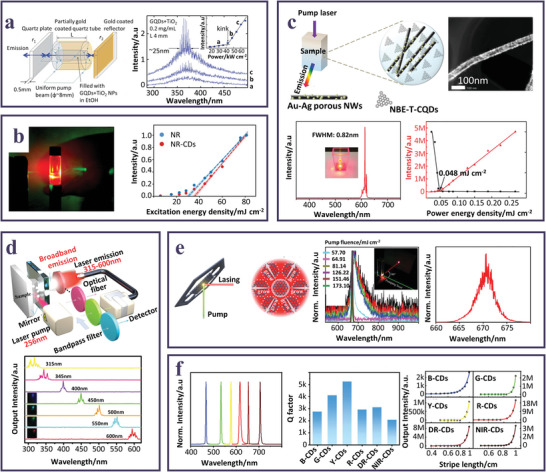
a) Sketch of a laser cavity consisting of a capillary quartz tube filled with a mixture of GQDs and TiO_2_ NPs; lasing spectra at increasing pumping fluence; in the inset light‐light curve. Reproduced and adapted with permission.^[^ ^71^ ^]^ Copyright 2013, The Royal Society of Chemistry. b) Picture of red RL emission of the NR‐CDs and TiO_2_ dispersion; light‐light curves for NR and NR‐CDs. Reproduced and adapted with permission.^[^ ^64^ ^]^ Copyright CC‐BY 4.0 2023, American Chemical Society. c) Sketch of the RL action from triangular‐shaped CDs and Au‐Ag bimetallic nanowire hybrid system; HAADF‐STEM of the Au‐Ag bimetallic nanowire; red RL emission spectra (inset picture of red RL emission); plots of the red integrated RL intensity and FWHM of the spikes in the RL spectra as a function of the pumping power (λ_ex_ = 532 nm). Reproduced and adapted with permission.^[^ ^76^ ^]^ Copyright 2018, WILEY‐VCH Verlag GmbH & Co. KGaA, Weinheim. d) Scheme of multicolor solid‐state laser optical setup; laser spectra of SiCDs/Et3BTC hybrids collected in the visible range through bandpass filters (inset: pictures of hybrid crystals observed behind different bandpass filters). Reproduced and adapted with permission.^[^ ^108^ ^]^ Copyright 2022, Wiley‐VCH GmbH. e) Scheme of laser generation mechanism in a sheet shaped CD assembly; normalized PL spectra of a solution of red emitting CDs at different pumping fluences (inset: picture of the laser red spot); laser emission spectrum of a CD solution at 57.7 mJ cm^−2^. Reproduced and adapted with permission.^[^ ^89^ ^]^ Copyright 2023, Wiley‐VCH GmbH. f) Normalized multicolored emitting CDs’ laser spectra, with Q‐factors and output emission intensity dependance on the variable excitation‐length. Reproduced and adapted with permission.^[^ ^79^ ^]^ Copyright 2023, Wiley‐VCH GmbH.

While random lasers based solely on GQD systems were pioneered, their efficiency and intensity remain relatively low. The modest optical gain produced can be mainly ascribed to the typical very small size of CDs, standing below 10 nm, which is not sufficient to induce an efficient scattering feedback mechanism. Indeed, to achieve RL the size of the scattering structures needs to be comparable to the pumped light wavelength. Thus, to enhance the gain efficiency, different larger nanomaterials can be added to the system to provide feedback by multiple scattering, and the process can be properly modulated by optimizing the scatterer's nature and concentration. In their work, Zhu and coauthors^[^ ^71^ ^]^ demonstrated that, by including TiO_2_ NPs with a mean size of ≈100 nm in the GQDs gain medium, the RL action was achieved at a lower threshold (**Figure** **5**a). The scatterers reduced the number of lasing modes for more resolved laser spectra, with power efficiency similar to those achieved by semiconductor lasers, demonstrating that the developed RL system exhibited performance compatible with the solid‐state lasing devices. NR‐derived CDs were used as gain media associated with 30 nm‐sized TiO_2_ NPs as passive scatterers in mirrorless RL experiments where the excitation beam was pumped through a cylindrical lens (Figure 5b).^[^ ^64^ ^]^ The emerging red RL emission was maximized by optimizing the CD and NP concentration, demonstrating narrower spectral bandwidth and lower thresholds than those exhibited by the molecular dye in the presence of TiO_2_ NP scatterers. The observed findings were ascribed to the structural features of CDs that act not only as gain media, but also contribute to the light scattering together with the TiO_2_ NPs.

The role of CDs as scattering agents in association with other fluorophores was also explored. Recently, a system where CDs were dispersed in an EG solution of Rhodamine B dye, exhibited RL with resonant feedback with sub‐nm resolved lasing modes in the spectral region of the dye.^[^ ^75^ ^]^ A further way to improve the performance of random lasers is enhancing the lasing output through plasmonic resonance by supporting gain medium with metallic nanostructures or rough surfaces acting as scattering centers. Thus, Liao et al.^[^ ^127^ ^]^ fabricated random closed‐loop cavities with coherent feedback taking advantage of the cooperative effect of light scattering and plasmonic interactions, developed through a system where the surface of vertically aligned UV light emitting gallium nitride nanorods was covered with CDs acting as scatterers also able to generate surface plasmon resonance due to the increased surface roughness, enhancing the UV fluorescence. More often, CDs were used as active gain medium in combination with plasmonic nanostructures. The development of a specifically designed gain medium able to conjugate the optical features of the carbon nanostructures with the plasmonic properties arising from Au‐Ag bimetallic nanowires resulted in highly efficient ultra‐stable coherent RL emission with a spectral tunability from blue to red.^[^ ^76^ ^]^ In their communication, Yuan et al. profited from the outstanding optical features of the solvothermally synthesized CDs, particularly of the minor bandwidths and the very small Stokes shifts, to achieve extremely narrow lasing emission at low pump thresholds, demonstrating even superior in comparison to the traditional random lasers based on perovskite or semiconductor NCs (Figure 5c).

Recently, a complex composite formed by a mixture of three nanomaterials was developed to obtain an intense single‐mode lasing.^[^ ^73^ ^]^ The designed optical active solution consisted of hybrids of highly concentrated DCM (4‐(dicyanomethylene)2‐methyl‐6‐(p‐dimethylaminostyryl)4H‐pyran) laser dye self‐assembled in nanowires, and fluorescent CDs aggregated on the DCM surface. A FRET process from DCM to CDs activated the RL emission in the deep red spectral region at the pump energy of 190.6 µJ pulse^−1^. The addition of TiN NPs as scatterers promoted the narrowing of the emission band, resulting in sharp peaks less than 1 nm wide at 472 µJ pulse^−1^ pumped threshold, demonstrating the coherent RL characterized by a Q‐factor of 2267.

While most of the literature concerning RL deals with solution‐based systems, only very few recent examples report lasing emission from solid‐state disordered systems, where fluorescent CDs were embedded in polymeric fibers,^[^ ^107^ ^]^ or included in hybrid crystals.^[^ ^108^ ^]^ Indeed, the fabrication of efficient and robust solid‐state laser devices requires addressing and overcoming issues related to aggregation‐induced quenching, instability due to continuous light pumping, and resistance to environmental moisture and oxygen, which can partially be resolved by incorporating nanostructures into matrices. Tunable wavelength and threshold RL were demonstrated from CD‐PS fibers showing excellent fluorescent stability under high temperature and environmental moisture conditions to achieve a multilevel anti‐counterfeiting system.^[^ ^107^ ^]^ A further application of solid‐state CD based gain medium in RL exploited grinded powdered white emitting hybrids of SiCDs and Et3BTC.^[^ ^107^ ^]^ Such hybrids were packed between a reflecting Al mirror and a dielectric mirror to form an optical cavity to improve the optical response and reduce the lasing threshold. Tunable RL emission wavelengths, ranging from near UV to red under 265 nm excitation, were obtained from the white light originating from the multiple fluorescent centers enclosed in the SiCD hybrids (Figure 5d). Sharp peaks of laser modes were observed over ≈60 kW cm^−2^ pumped threshold from different detection angles and stable solid‐state laser radiation was detected at higher temperatures.

In some cases, owing to the specific surface chemistry and consequent optical properties of certain types of CDs, strong aggregation can be induced by variations in pH. The protonation of surface functional groups can cause the spread of the delocalized π‐electronic system, resulting in a bathochromic shift of fluorescence up to the red or deep‐red spectral range, occurring without aggregation‐induced quenching events. In a recent work, Lu et al.^[^ ^89^ ^]^ demonstrated that the acidification of CDs obtained by the solvothermal treatment of pyrogallol caused their aggregation in sheet‐like architectures and the fluorescence shift to the deep‐red region (Figure 5e). These crystalline structures acted as a resonant cavity, as increasing pumping intensity over 57.7 mJ cm^−2^, the emission bandwidth narrowing, and ASE observation were followed by a multimode RL orthogonally to the excitation beam.

Besides achieving optical tunability in RL, another difference from classical lasers lies in the greater difficulty of obtaining single‐mode lasing. In RL, how the pumping beam evolves during scattering and amplification affects the propagation of modes, and consequently, the effects due to interference construction need to be considered. Interestingly, recent literature reports an example of multi‐colored emitting CDs vertically pumped in a solution‐processed laser device showing either ASE or single‐mode RL emission, according to the pumped threshold.^[^ ^89^ ^]^ In their work, Lu and co‐authors achieved RL in a wide spectral range, from blue to NIR region, observing narrow band emission, laser thresholds varying from ≈11 to 320 mJ cm^−2^, and strong monomodal emission characterized by high Q‐factors, significant gain coefficients, and superior stability in comparison to laser devices based on the common commercial organic dyes (Figure 5f). In addition, such systems were successfully employed to fabricate high‐quality speckle‐free laser imaging and dynamic holographic display, and the work is one of the very few examples where the CD‐based RL was exploited in a technological application.

In conclusion, several types of laser cavities have been explored using CDs as active materials. The choice of the cavity type is not always directly linked to the specific optical properties of the CDs, but often depends on the origin of the CD emission and to their relationship to the physical state of the nanoparticles and the possible applications. For CDs in solution, the Fabry‐Perot cavity, consisting of two‐faced mirrors, is the most straightforward for testing laser emission in the lab. DFB lasers, which incorporate a grating, are suited for CDs with broader luminescence linewidths, allowing for emission tunability. DBR and VCSEL are ideal for solid‐state applications, such as CDs embedded in polymers or powders, as they facilitate cavity miniaturization and better control over laser mode selection and divergence. RL benefits from CDs serving as scattering media, increasing the efficiency of the laser action. WGMs offer a quick and elegant method for achieving laser action using pre‐formed microresonators, of particular interest for sensing.

**Table** **3** provides a summary of the main lasing modes of CDs, including the spectral range, Q factor values and the thresholds achieved in the recent literature.

**Table 3:** Survey of CDs‐based lasing operating modes and related lasing properties.

Lasing operating mode	Excitation source	Lasing spectral range [nm]	Q‐factor	Lasing threshold [kW cm^−2^]	Year^[Ref.]^
Cylindrical microcavity: CD‐coated optical fibre	Nd:YAG λ = 266 nm	Multiple lasing peaks at 390 – 420	1300	120	2012^[^ ^70^ ^]^
Quartz cuvette CDs deposited on quartz substrate	Nd:YAG λ = 266 nm	Wide spectrum 350–650 nm	n.d.	210	2012^[^ ^72^ ^]^
Cylindrical quartz tube with half surface Au coated, inserted into a parallel mirror F‐P cavity	Nd:YAG λ = 266 nm	≈360 nm	n.d.	30	2013^[^ ^71^ ^]^
		≈360 nm	n.d.	180	
Cylindrical quartz tube with inner capillary filled with CDs in epoxy, inserted in a plane parallel DBR‐grating F‐P cavity, eventual addition of a ball lens	Nd:YAG/OPA λ = 450 nm	494–554 nm	n.d.	200	2014^[^ ^109^ ^]^
Quartz cuvette with front and back face coated with DBR mirrors with 95 and 99% reflectance	Nd:YAG λ = 355 nm	560 nm	n.d.	274	2014^[^ ^80^ ^]^
Quartz cuvette CDs embedded in PI and deposited on glass substrate	Nd:YAG λ = 355 nm	460 nm	n.d.	120 350 ≈600	2016^[^ ^78^ ^]^
RL in solution	Nd:YAG λ = 532 nm	587 nm	n.d.	1260	2017^[^ ^93^ ^]^
CDs in NaCl crystals – WG mode	Ti:Sapph/OPA, λ = 360 nm	514 nm	447	n.d.	2017^[^ ^111^ ^]^
CD film sandwiched between a quartz plate and a dielectric mirror	Nd:YAG λ = 355 nm	400–500 nm	n.d.	15	2017^[^ ^104^ ^]^
Quartz cuvette	Nd:YAG/OPA λ = 355 nm	476 nm	n.d.	17.4	2019^[^ ^76^ ^]^
	Nd:YAG/OPA λ = 480 nm	520 nm	n.d.	10.4	
	Nd:YAG/OPA λ = 532 nm	613 nm	n.d.	9.6	
WGM in an optical fibre coated with CDs	Nd:YAG λ = 532 nm	580–590 nm	3600	12	2019^[^ ^112^ ^]^
Vertical optical cavity formed by Ta_2_O_5_/SiO_2_ DBR dielectric stacks	Nd:YAG λ = 355 nm	500–600	798.4	7.2	2019^[^ ^106^ ^]^
Solid state cavity formed by two DBR mirrors and a SiO_2_ spacer, CDs injected in solution	Nd:YAG λ = 532 nm	643.9	4600	1850	2021^[^ ^77^ ^]^
Planar microcavity formed by an aluminium and dielectric mirror interspaced with a CD–ormosil gel hybrid	Nd:YAG λ = 532 nm	570–605	n.d.	≈0.07	2021^[^ ^105^ ^]^
RL in solid‐state CD/PS composite fibres	Nd:YAG λ = 355 nm	428–560	n.d.	800–4167	2021^[^ ^107^ ^]^
Solid‐state planar F–P microcavity formed sandwiching CD film between an Al mirror and quartz substrate	Nd:YAG λ = 532 nm	580–620	n.d.	10–33	2021^[^ ^110^ ^]^
SiCDs/Et3BTC hybrids sandwiched between an Al mirror and dielectric mirror	Nd:YAG/OPA λ = 265 nm	315–600	n.d.	60	2022^[^ ^108^ ^]^
Quartz cuvette	Nd:YAG λ = 355 nm	467.3	2748.8	2691	2023^[^ ^79^ ^]^
	Nd:YAG λ = 355 nm	533.5	4103.8	5127	
	Nd:YAG λ = 532 nm	577.4	5249.1	7616	
		616.3	2920.5	1586	
		653.5	3111.9	6271	
		705.1	2073.8	2554	
Planar microcavity formed by P‐CDs between two DBR	Nd:YAG λ = 355 nm	442	n.d.	1.6	2023^[^ ^103^ ^]^
Multi‐mode RL, generated by the sheet shape structure of the R‐CDs that is likely to play a role similar to a resonant cavity	Nd:YAG λ = 532 nm	670	n.d.	n.d.	2023^[^ ^89^ ^]^
RL in hybrids CDs/Et3BTC, for anti‐counterfeiting	Nd:YAG λ = 355 nm	≈450	n.d.	n.d.	2023^[^ ^113^ ^]^
Quartz cuvette inserted in a macroscopic F‐P cavity with external spherical/flat mirrors or grating	Nd:YAG/OPA λ = 535 nm	630	n.d.	130	2023^[^ ^64^ ^]^
Cylindrical glass vial containing CDs and scattering TiO_2_ NPs			n.d	6000	
Planar FP in a 2 mm quartz cuvette and cylindrical lens	Nd:YAG λ = 355 nm	450‐460	5853	2758–5693	2023^[^ ^87^ ^]^
Planar F‐P cavity formed by two DBR mirrors interspaced by a plastic spacer	OPA λ = 450 nm	490–500	n.d.	41 000	2024^[^ ^114^ ^]^

## 5 Applications

The rapid evolution toward miniaturization, portability, and integration of solution‐processable lasers is a hallmark of technological research in the last decade, driven by features such as facile spectral tuning, straightforward chemical processing, low operational thresholds, and inherent mechanical flexibility. Miniaturized lasers play indispensable roles across various domains, including information storage, communication systems, micro/nano processing, medicine, and biosensing. The emergence of solid‐state micro/nano lasers utilizing innovative gain materials like organic semiconductors, colloidal QDs, lead halide perovskites NCs, and CDs paves the way for groundbreaking advancements. These materials boast distinct advantages, such as solution processability, and thus easy fabrication and functionalization, spectral tunability, and flexibility, that were pointed out, making them easily integrable into various technological applications. The specific characteristics of CD‐based lasers make them ideal for photonics where the demand for compact, efficient, and versatile solid‐state lasers is steadily escalating.^[^ ^128^ ^]^ However, despite the large interest in carbonaceous NPs as active gain materials in innovative lasing systems, potentially appealing in various fields such as optical communications, colorful imaging, identification technologies, or biological counting, CD laser performances are still far from being effectively applied in real devices and only very few real application were demonstrated, mainly for RL systems.

Recent literature identifies two primary applications for CD laser: anticounterfeiting and the holographic displays.

Concerning the security and authentication field, thanks to their distinctive optical properties and the specific fingerprints of the RL, CD‐based lasers can offer versatile utility as anti‐counterfeiting labels, optical tags, and encrypted data encoding tools. The distinctive optical signatures of CDs serve as reliable markers for verifying the authenticity and integrity of various items, including products, documents, and digital information. Additionally, the intrinsic low toxicity of the carbonaceous nanoparticles enables their use in widely accessible environments and in portable systems.

**Figure 6:**
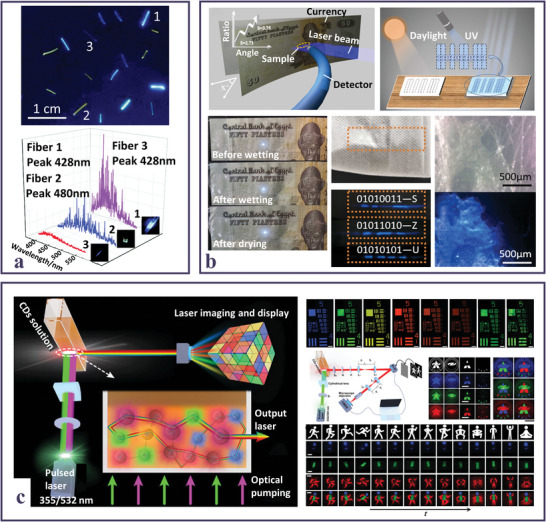
a) Picture under UV light of CD‐fibers; and corresponding emission spectra detected under 370 nm laser excitation. Reproduced and adapted with permission.^[^ ^107^ ^]^ Copyright 2021, The Royal Society of Chemistry. b) Illustration of the optical setup of the multi‐level anticounterfeiting strategy based on flexible fabrics blended with CD/Et3BTC hybrids; scheme of the encoding technique with the hybrids; photographs of the hybrids blended within banknotes before and after wetting, and after drying; photographs of the hybrid pattern blended with the fabric under ambient illumination; picture of the encode sequences under UV‐light; micrographs of the fabric blended with hybrids under ambient and UV light. Reproduced and adapted with permission.^[^ ^113^ ^]^ Copyright 2023, Elsevier B.V. c) Artwork of colorful laser imaging and holographic display fabricated with full color emitting CD based lasers; optical images of resolution test charts of multi‐color emitting CD based lasers and commercial laser sources (scale bars, 100 µm); experimental setup of holographic displays; examples of holographic static or dynamic displays (scale bars, 1 mm) using CD lasers as light source. Reproduced and adapted with permission.^[^ ^79^ ^]^ Copyright 2023, Wiley‐VCH GmbH.

In,^[^ ^107^ ^]^ PS fibers incorporating blue‐emitting CDs with enhanced fluorescence stability under challenging environmental conditions (high temperature and high humidity) were used as effective gain media in RL. The authors demonstrated that, by controlling both the time duration of the thermal decomposition of PS before the thread drawing and the fiber diameters, the lasing threshold and peak wavelength of the RL modes could be tuned to obtain a multilevel anti‐counterfeiting system (**Figure** **6**a), thus significantly enhancing the security. The ability to design a multilevel system can have potential applications in information storage, encoded bar codes, identification tags, and anti‐counterfeiting labels for commercial products.

More recently the same authors proposed a significant advancement in flexible anti‐counterfeiting techniques, providing valuable insights to foster further developments in this field.^[^ ^113^ ^]^ CDs and Et3BTC were explored as two distinct gain media for achieving lasing in the UV‐B (325 nm) and visible (450 nm) spectra, respectively. The responses of these two RL media under different conditions exhibited notable differences, enabling the tuning of lasing peak intensities by adjusting parameters such as working temperature, excitation area, and substrate bending angle. Consequently, a novel strategy was suggested, which involves the modulation of dual RL media along with a flexible substrate (Figure 6b). This approach is particularly suitable for enhancing the security of high‐value items such as banknotes, artworks, and commodities, providing advanced security features that are difficult to replicate.

Computer‐generated holography (CGH) holds great potential for various applications, spanning from direct vision to virtual and augmented reality, as well as automotive display systems. Despite significant advancements in holographic display research, the image quality and safety for human eyes are inherently constrained by speckles introduced by coherent light sources. Addressing these challenges, Wetzstein et al.^[^ ^129^ ^]^ pioneered a method utilizing a random laser for generating CGH, showcasing enhanced speckle properties in the resulting holograms compared to those generated using coherent lasers. Their approach yielded crisp, high‐contrast 2D and 3D images that are not only bright and safe for viewing but also nearly devoid of speckle artifacts.

Recently, speckle‐free optical images were achieved by exploiting full‐color emitting CDs, including bright blue, green, yellow, red, deep‐red, and NIR variants as light sources in RL.^[^ ^79^ ^]^ The optical images exhibited higher quality, namely high speckle contrast and low contrast‐to‐noise ratio, and superior light distribution compared to those captured using commercial lasers (Figure 6c). The potential for full‐color laser imaging and display applications was demonstrated, thus significantly promoting the practical applications of solution‐processable CD‐based lasers in the fabrication of advanced devices. By leveraging the advantages of highly monochromatic and coherent laser emission, laser display technology has the potential to revolutionize the conventional display industry, thanks to its expansive color gamut, vivid color saturation, and impressive contrast levels. Zhang et al.^[^ ^79^ ^]^ also introduced a groundbreaking approach by developing a periodic full‐color pixelated microlaser array. Each set of red, green, and blue (RGB) microlasers constituted a pixel within this array, enabling the creation of a self‐emitting panel for display applications (Figure 6c). By manipulating the excitation mode on a single pixel, the authors obtained flexible laser output with tunable wavelengths spanning nearly the entire visible spectrum. This capability offers significant advantages for dynamic displays characterized by high color saturation.

The potential presented by this fabrication method for achieving wide‐span full‐color lasers, particularly those with NIR emission, holds significant promise. This is particularly noteworthy given the extensive applications of NIR lasers in space optical communication, night vision, and especially in clinical imaging and therapy.

## 6 Potential, Challenges and Perspectives

Although there are still very few implementations of CD‐based lasers in effective devices and systems, delving deeper into their technological potential we can envision their applications across various fields.

Among them, lasers are highly efficient in information storage.^[^ ^130^ ^]^ Zhao et al.^[^ ^131^ ^]^ developed an ordered single‐mode microlaser array with a stimulus‐response tailored for information storage and encryption. Such technology establishes a robust platform for high‐security information protection that could be realized using CD‐based microresonators.

Also, highly monochromatic and coherent lasers can be applied to single‐photon optical switches that could replace photonic transistors in the next generation of computers.^[^ ^132^ ^]^ The single‐photon optical switch consisted of DBR microcavities based on π‐conjugated ladder polymers. Shining two pulsed laser beams (a pump beam and a seed beam) into the microcavity and switching between them by setting their state to “‘0′” or “‘1′” fulfilled the function of an optical transistor (photon switch). These photon switches provided substantial amplification and sub‐picosecond switching times. In addition to direct power savings, the switch does not require cooling and is 100 to 1000 times faster than the current best commercial transistors.

The CD‐based lasers are also expected to strongly impact the biological domains, like human health, homeland security, environmental monitoring, and diagnostics, where there is a pressing demand for portable systems capable of detecting and characterizing individual pathogens, and other nanoscale objects without relying on markers. However, minute size and low refractive index contrast of most nanoscale objects make them exceedingly challenging to detect without labeling. Platforms based on micro‐, or nano‐photonics emerged as highly sensitive solutions for such applications, leveraging the combination of high Q‐factor and small volume to significantly enhance light‐matter interactions.^[^ ^133^ ^]^ Therefore, sensors that utilize CD‐based micro‐ and nano‐lasers allow for detecting objects beyond the reach of passive resonator sensors. The rapid progress in biophotonics and biomedical sciences pointed out the need for photonic structures that can interface with biological systems, manipulating light for sensitive signal detection and precise cellular imaging.^[^ ^111^, ^133^, ^134^ ^]^ Conventional structures based on artificial materials often pose compatibility issues. In this perspective the advances in CDs along with their distinctive optical properties, biocompatibility, and biodegradability highlight their unique possibility as probes for bio‐detection and imaging, mainly covering three key systems biological lasers, cell‐based waveguides, and biomicrolenses, and outline future opportunities and potential improvements.

Interestingly, proteins can also be used as a gain medium, offering versatile applications in cell counting, imaging, intracellular sensing, ultra‐sensitive biomolecule detection, and photo‐based cancer cell therapies.^[^ ^135^, ^136^ ^]^ In 2011, Gather and Yun demonstrated the first biological laser implementation within a single spherical living cell using green fluorescent protein as the gain medium.^[^ ^137^ ^]^ This seminal work showcased cells as large as 15 µm placed in a DBR microcavity, forming a biological laser. This research opens new avenues for the potential widespread use of non‐toxic and highly biocompatible CD‐based lasers incorporated in living cells.

Artificial smart skin with its flexible components, replicating and improving human skin function, can be extremely valuable in health monitoring, human‐computer interaction, augmented reality, prosthetics, and bionic robotics. Zhao et al.^[^ ^138^ ^]^ reported the design and construction of artificial photonic skin based on flexible organic micro‐nano laser arrays. They fabricated 3D‐supported organic microdisk structures on a flexible polymer substrate to achieve single‐mode laser output, significantly enhancing the recognition and accuracy of sensing signals. This formed the basis of a sensor unit responsive to the deformation of a flexible substrate, which was used to detect human motion and recognize various gestures. This new flexible photonics chip has wide potential applicability in human sensory reconstruction, human‐computer interaction, and robot self‐protection systems.

However, it is important to note that the viability and feasibility of most of these applications still require full demonstration, thus necessitating a thorough assessment of their anticipated potential. In most cases, the reproducibility issues related to the synthesis of CDs, the difficulty in designing a priori the optical properties of the carbonaceous nanoparticles, the large spectral bandwidths and the still improvable QY can limit their exploitation in technologically relevant applications. QY values reported in the literature are commonly in the range of 40%–80% although QYs above 95% have recently been demonstrated (Tables 1 and 2). Despite their potential, existing CD lasers still cope with challenges related to spectral purity and stability, failing to achieve a single longitudinal mode with a high signal‐to‐noise ratio. In addition, often the laser threshold of CDs is still too high, while the pursuit of high‐performance laser emission hinges on the development of CDs with elevated QYs. In this perspective, specific challenges need to be tackled. Efforts to narrow the laser bandwidth, increase the emission efficiency and design and engineer optical cavities to reduce the laser threshold of CDs are crucial in this regards.^[^ ^87^ ^]^

Despite the current limitations and considering the relatively recent discovery of CDs, significant strides have been made in recent years to enhance the properties of these fascinating nanomaterials that hold invaluable properties of low toxicity and biocompatibility, paving the way for laser miniaturization in shaping the future of integrated lighting devices.

## Conflict of Interest

The authors declare no conflict of interest.
